# Human mandibular shape is associated with masticatory muscle force

**DOI:** 10.1038/s41598-018-24293-3

**Published:** 2018-04-16

**Authors:** Tanya Sella-Tunis, Ariel Pokhojaev, Rachel Sarig, Paul O’Higgins, Hila May

**Affiliations:** 1 Department of Anatomy and Anthropology, Sackler Faculty of Medicine, Tel Aviv University, Ramat Aviv, Tel Aviv 69978 Israel; 2 Shmunis Family Anthropology Institute, Dan David Center for Human Evolution and Biohistory Research, The Steinhardt Museum of Natural History, Sackler Faculty of Medicine, Tel Aviv University, Ramat Aviv, Tel Aviv 69978 Israel; 3 The Maurice and Gabriela Goldschleger School of Dental Medicine, Sackler Faculty of Medicine, Tel Aviv University, Ramat Aviv, Tel Aviv 69978 Israel; 4 Centre for Anatomical & Human Sciences, Department of Archaeology and Hull York Medical School, University of York, Heslington, York, YO10 5DD UK

## Abstract

Understanding how and to what extent forces applied to the mandible by the masticatory muscles influence its form, is of considerable importance from clinical, anthropological and evolutionary perspectives. This study investigates these questions. Head CT scans of 382 adults were utilized to measure masseter and temporalis muscle cross-sectional areas (CSA) as a surrogate for muscle force, and 17 mandibular anthropometric measurements. Sixty-two mandibles of young individuals (20–40 years) whose scans were without artefacts (e.g., due to tooth filling) were segmented and landmarked for geometric morphometric analysis. The association between shape and muscle CSA (controlled for size) was assessed using two-block partial least squares analysis. Correlations were computed between mandibular variables and muscle CSAs (all controlled for size). A significant association was found between mandibular shape and muscle CSAs, i.e. larger CSAs are associated with a wider more trapezoidal ramus, more massive coronoid, more rectangular body and a more curved basal arch. Linear measurements yielded low correlations with muscle CSAs. In conclusion, this study demonstrates an association between mandibular muscle force and mandibular shape, which is not as readily identified from linear measurements. Retrodiction of masticatory muscle force and so of mandibular loading is therefore best based on overall mandibular shape.

## Introduction

The influence of masticatory muscle action on the development of craniofacial morphology has received considerable attention in the dental literature (see review article by Pepicelli *et al*.^1^). Since bone adapts to loads by remodeling to reach the optimal form to withstand them (Wollf’s law)^2^, it has been hypothesized that craniofacial skeletal form is largely determined by mechanical loading (e.g.^3–6^). This has been supported by many clinical and experimental studies. Thus, an association exists between muscle cross-sectional areas, which are approximately proportional (excluding pinnate muscles) to force generation, and craniofacial morphology, as found by studies using a range of methodological approaches (e.g., finite elements, CT models, strain gauges)^7–12^. Accordingly, it was established that facial types are associated with bite force, i.e. brachycephalic pattern with strong bite force and dolichocephalic with weak bite force^7,13,14^. Experimental studies show that the decreased functional demands on mandibles of animals fed a soft diet results in structural changes in the masticatory muscles^15^, as well as morphological alterations of the mandible, such as reduced size of the alveolar bone^16–18^.

Mandibular form and development have been extensively studied (e.g.^19,20^). Yet, how common measurements of human mandibular morphology and size covary with masticatory muscle forces has not been investigated in detail. This is a significant shortcoming for clinicians and anthropologists alike, since knowledge of how masticatory muscle force and mandibular form covary could enable the latter to be used to reconstruct diet and food preparation techniques in ancient populations. Although several studies have shown associations between craniofacial and mandibular shape and different feeding strategies^21–24^, efforts to reveal dietary habits and food preparation techniques from the oral apparatus have focused mainly on the study of oral pathologies such as caries, periodontal diseases, ante-mortem tooth loss, and attrition^25,26^.

The current study was therefore carried out to gain greater insight into the associations between muscle forces and mandibular morphology. Such a study requires living individuals and is best established using computerized tomography (CT) scans in which bone and soft tissue shadows are visible. More so, muscle cross-sectional areas (CSA) from CT, magnetic resonance imaging and ultrasound scans can be used as a surrogate for the peak forces that can be generated by the masticatory muscles^7,9,11,12,27–33^.

The aims of this study were to identify associations between masticatory muscle force (as estimated by CSAs) and mandibular shape and to relate variations in specific muscle CSAs (masseter and temporalis) to specific aspects of mandibular shape and size variation. Two hypotheses were tested: H0_1_ - no association exists between the CSAs of the masseter and temporalis muscles and mandibular shape; H0_2_ - no associations exist between temporalis and masseter CSAs and anthropometric (linear and angular) measurements of the mandible. The first hypothesis was examined using shape variables derived from landmark data. The second hypothesis was tested using Pearson correlations to assess relationships between muscle CSAs and mandibular variables. The second analysis was carried out for practical reasons since archeological mandibles are sometimes too fragmented to readily allow shape analysis.

## Material and Methods

The study included 382 individuals (193 males and 189 females) aged 18–80 years who had undergone a head and neck CT scan at Carmel Medical Center, Haifa (Brilliance 64, Philips Medical System, Cleveland, Ohio: slice thickness 0.9–3.0 mm, pixel spacing 0.3–0.5 mm, 120 kV, 250–500 mAs, number of slices 150–950 and Matrix 512*512), between the years 2000 and 2012. All CT scans were carried out for diagnostic purposes, where a CT scan was medically necessary. Inclusion criteria were as follows: age between 20 and 80 years, intact lower incisors, and at least two teeth of the posterior unit (premolars and/or molars) on each side. Exclusion criteria included the absence of the lower incisors; dental implants and metal restorations that interfere with imaging and so, measurement; prominent facial and mandibular asymmetry; craniofacial, temporomandibular joint, or muscular disorders; trauma; previous surgery on the head and neck region (based on medical files or signs on the skull); and technically aberrant CT scans. This study was approved by the ethical board of the Carmel Medical Center, Israel (number: 0066-11-CMC) and followed their guidelines.

### Evaluating muscle areas (Force)

**Figure 1:**
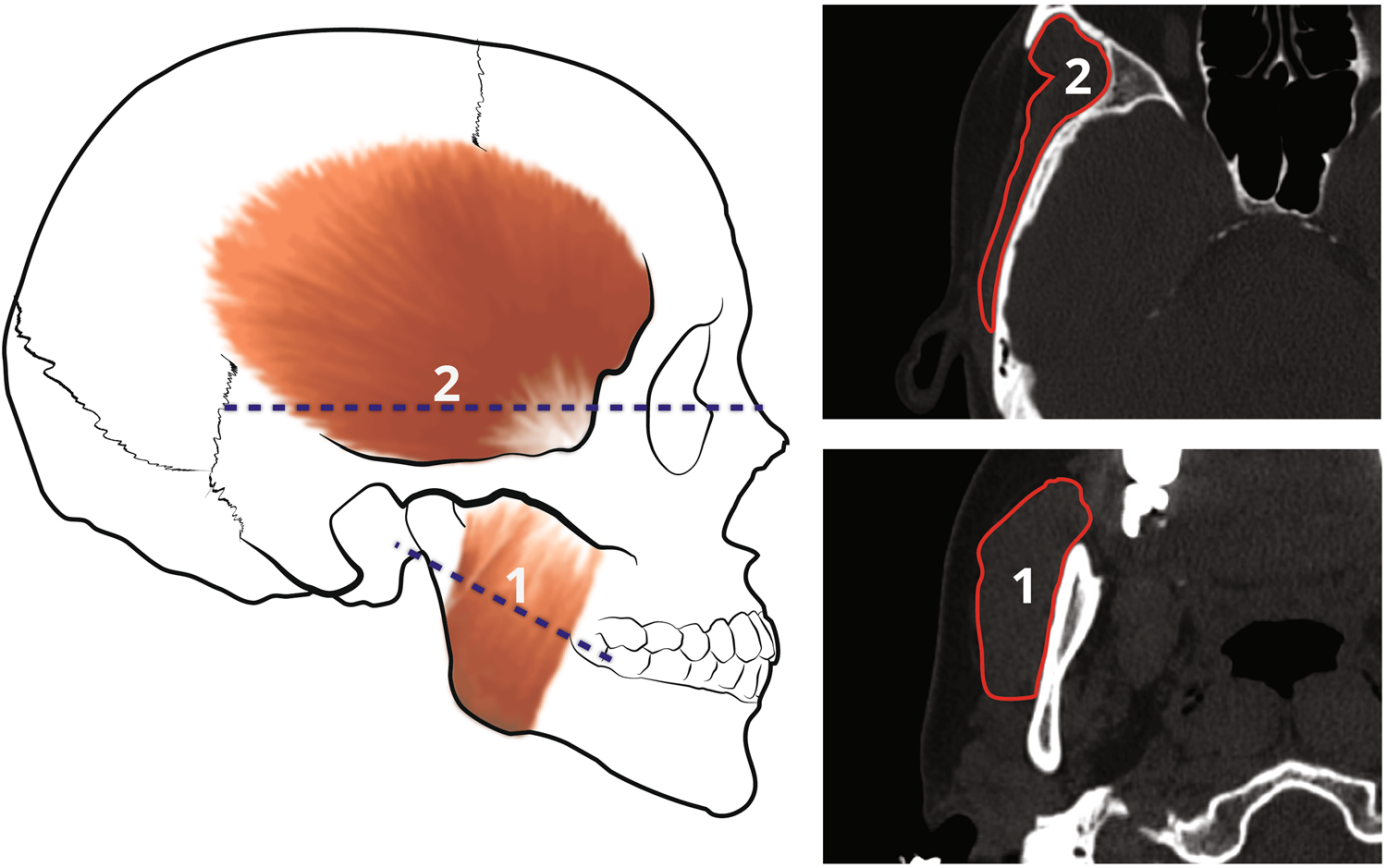
Muscle cross-sectional area measurement following Weijs and Hillen^28^. Masseter (1) area was estimated by tracing it on the CT scan sectioned 3 cm ventro-cranially to the jaw angle, 30° relative to the Frankfurt horizontal plane. Temporalis (2) area was measured one cm cranially to the zygomatic arch, parallel to the Frankfurt horizontal plane.

CSAs of the masseter and temporalis muscles (which reflect peak force) were measured using the planar mode for sectioning CT stacks, and the ‘region of interest’ tool for tracing outlines and measuring areas available on the Brilliance Workspace Portal (Philips v. 2.6.1.5). Masticatory muscle CSAs were measured following the method of Weijs and Hillen^28^ (Fig. 1). The muscle CSA was controlled for mandibular size using either mandibular centroid size (in GM analyses) or the geometric mean of the mandibular linear measurements (MGM - for analyses of anthropometric data; see statistical analysis section).

### Evaluating mandibular shape using the geometric morphometrics

**Figure 2:**
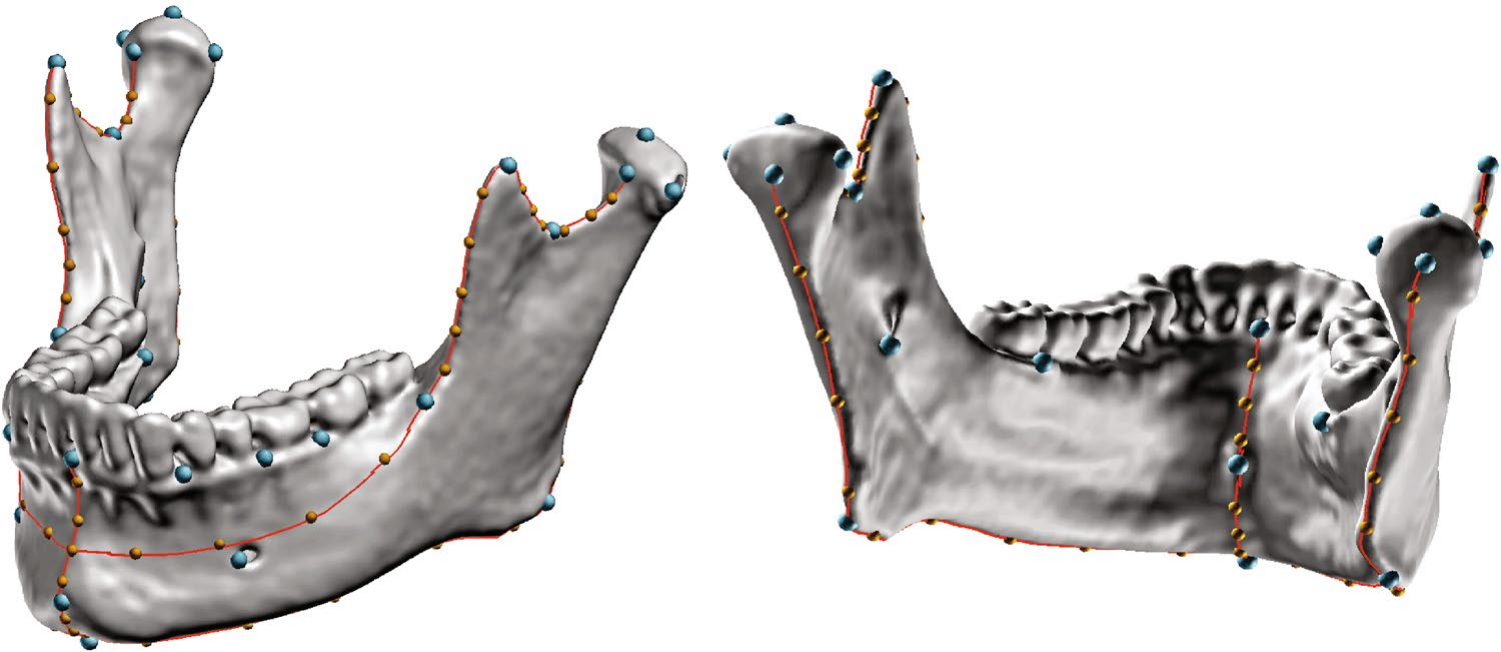
Landmarks (blue), curves (red) and curve semi-landmarks (yellow) placed on a 3D surface mesh of a mandible, see Tables 1 and 2 for definitions.

62 mandibles (30 males and 32 females) were segmented and reconstructed from the CT stacks using Amira (v6.1). Semi-automated segmentation of CT sections was carried out based on grey level thresholds. Manual refinement of segmentation was carried out where needed. The inclusion criteria for this group were: age 20–40 years to control for age effect on muscle CSAs and CT scans with no artefacts that may interfere with the segmentation (e.g., tooth filling and dental crown). The 3D form of the mandible was characterized using 35 landmarks and 60 curve semi-landmarks (representing 13 curves; Tables 1 and 2; Fig. 2). The landmarks, curves and curve semi-landmarks were placed on the mandibular surface mesh using the EVAN Toolbox software (v.1.71) and semi-landmark sliding was carried out to minimise bending energy^34^.

**Table 1:** Definition of landmarks placed on the mandibular surface.

Landmark		Definition
1	Gnathion	The inferiormost point of the mandibular body in the midsagittal plane
2	Infradentale anterior	The anteriormost point of the mandibular alveolar border in the midsagittal plane
3	Linguale	The genial tubercle
4	Infradentale posterior	The postero-superior point of the mandibular alveolar border in the midsagittal plane
5	Pogonion	The anteriormost point in the midsagittal plane
6+7	C-P3	The anteriormost point between canine and 1^st^ premolar (left and right, respectively)
8+9	P4-M1	The anteriormost point between 2^nd^ premolar and 1^st^ molar (left and right, respectively)
10+11	M1-M2	The anteriormost point between 1^st^ and 2^nd^ molars (left and right, respectively)
12+13	Mental foramen	The anteriormost point of mental foramen (left and right, respectively)
14+15	Root of ramus	The anteriormost point of the ramus rim at the level of the alveolar ridge (left and right, respectively)
16+17	Gonion	The point on the projection of the bisection of the mandibular angle (left and right, respectively)
18+19	Lateral condyle	From a superior view, the lateralmost point of the condyle (left and right, respectively)
20+21	Center of condyle	From a superior view, the central point of the condyle (left and right, respectively)
22	Medial condyle	From a superior view, the medialmost point of the condyle (left and right, respectively)
24+25	Sigmoid notch	The inferiormost point of the mandibular notch, when the mandible is positioned in the mandibular plane (left and right, respectively)
26+27	Coronion	The superiormost point of the coronoid process (left and right, respectively)
28+29	Mandibular foramen	The inferiormost point of the mandibular foramen (left and right, respectively)
30+31	Alveolar process - lingual aspect	From a superior view, the intersection between a line tangent to the lingual alveolar process of the molar teeth and a line, perpendicular to it, passing through the ramus root (left and right, respectively)
32+33	Anterior condyle	The anterosuperior point of the mandibular notch (left and right, respectively)
34+35	Posterior condyle	The posteriormost point of the condyle at its center (left and right, respectively)

**Table 2:** Definitions of curves placed on the mandibular surface and number of curve semi-landmarks (sLM).

Curve		Definition	# of sLMs
1**+**2	Mandibular body (left and right)	Passing from the Ramus root (LMs 14/15) along an oblique line to the midheight of the mandibular symphysis	8
3**+**4	Anterior rim of ramus (left and right)	Passing from coronion (LM 26/27) to ramus root (LM 14/15)	10
5**+**6	Inferior margin of mandibular body (left and right)	Passing from Gonion (LM 16/17) to Gnathion (LM 1)	10
7**+**8	Posterior rim of ramus (left and right)	Passing from posterior condyle (LM 34/35) to gonion (LM 16/17)	10
9**+**10	Mandibular notch	Passing from anterior condyle (LM 32/33) to coronion (LM 26/27) on the superior border of the mandibular notch	10
11	Anterior symphysis	Passing from infradentale (LM 2) to pogonion (LM 5) in the midsagittal plane	3
12	Inferior symphysis	Passing from pogonion (LM 5) to linguale (LM 3) in the midsagittal plane	6
13	Posterior symphysis	Passing from linguale (LM 3) to orale (LM 4) in the midsagittal plane	3

### Evaluating mandibular shape and orientation using linear and angular measurements

**Figure 3:**
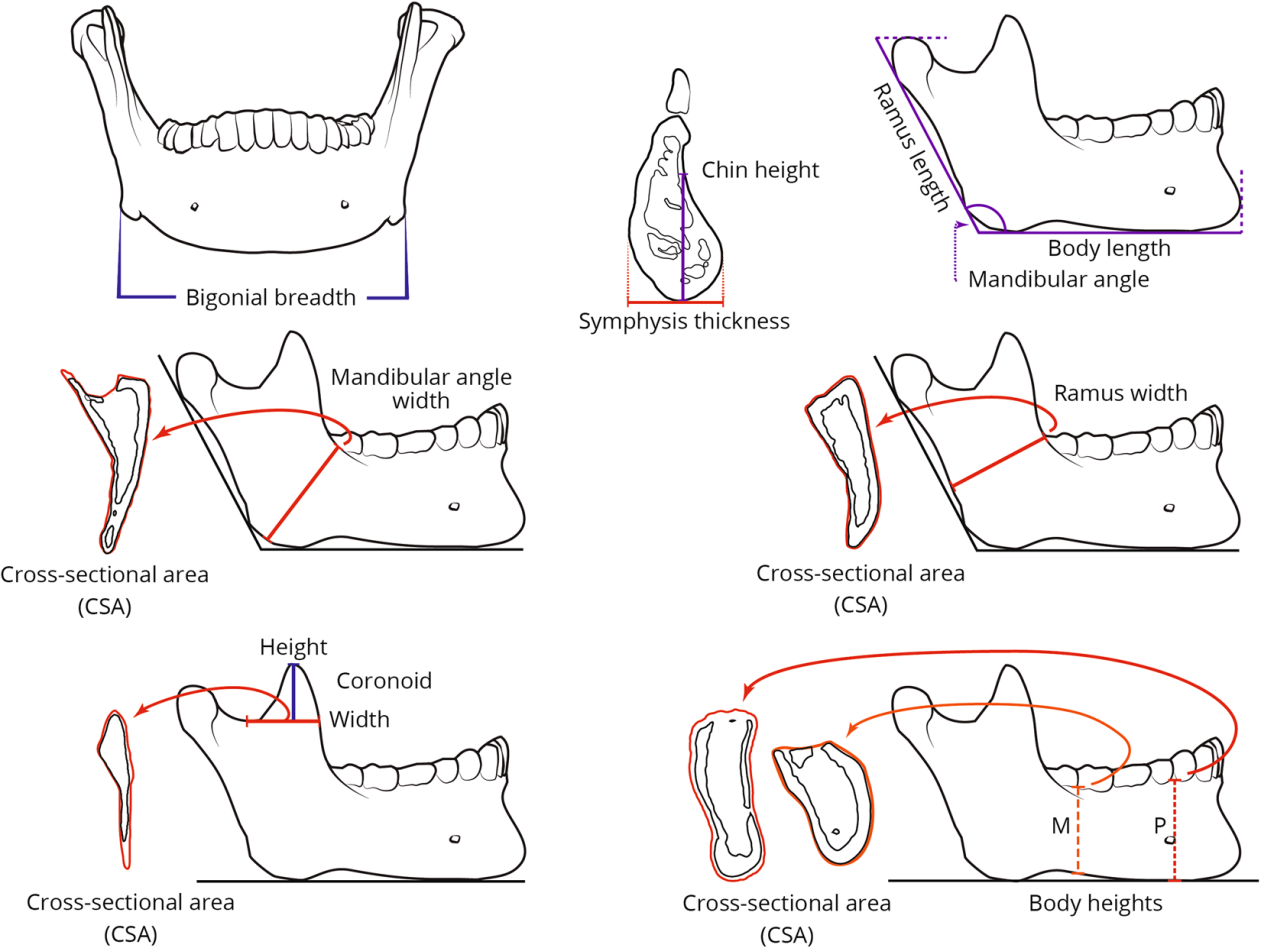
Linear, angular and cross-sectional area measurements of the mandible, see Table 3 for definitions.

17 linear, CSA and angular measurements of the mandible were obtained. These include traditional measurements and non-standard ones that are feasible due to the use of CT scans^35^ (Table 3; Fig. 3). All measurements were taken directly from CT scans using the Brilliance Workspace Portal (Philips v. 2.6.1.5). All linear measurements were controlled for mandibular size using the MGM (square roots of the CSA measurements of the mandible were divided by the MGM) following the principles presented in Jungers *et al*.^36^. This accounts for the effects of general size when assessing how the resulting indices covary with muscle CSAs, also scaled for MGM.

**Table 3:** Linear, angular and cross-sectional area (CSA) measurements of the mandible.

Measurement	Definition
Bi-gonial breadth	Distance between right and left gonion
Mandibular angle	The angle formed by the inferior border of the mandibular body and the posterior border of the ramus
Mandibular angle width	The distance between the gonion and deepest point on the concavity connecting the anterior border of the ramus with the mandibular body
Mandibular angle width CSA	The cross-sectional area of the mandibular body along the mandibular angle width line
Ramus length	The distance from the highest point on the condyle to the gonion
Ramus width	The distance between the anterior and posterior indentations of the mandible ramus
Ramus width CSA	The cross-sectional area of the mandibular ramus along the ramus width line
Coronoid width	The distance between the deepest point on the mandibular notch and the anterior border of the coronoid process
Coronoid width CSA	The cross-sectional area of the mandibular ramus along the coronoid width line
Coronoid height	The vertical distance between the most superior point of the coronoid process and the coronoid process width line, perpendicular to it
Mandibular body length	The distance from the most anterior point of the chin to a line placed along the posterior border of the ramus
Mandibular body height (P1-P2 and M2-M3)	The vertical distance from the alveolar crest between the 1st and 2nd premolars, or distal to the 2nd mollar, to the inferior border of the mandibular body
Mandibular body CSA (P1-P2 and M2-M3)	The cross-sectional area of the mandibular body along the body height line
Symphysis thickness	In the midsagital plane, the distance between the Pogonion and the most posterior point of the symphysis
Chin height	The distance between the menton and the deepest point of the concavity between the posterior infradentale and pogonion

### Statistical analysis

Statistical analyses of landmarks, indices and angular measurements were carried out using PAST (v. 3.15) or SPSS (v.22.0). The threshold of significance was taken as p = 0.05 in this study.

### Landmark based analyses

*Reliability:* Intra- and inter-observer variation in the shape of mandibular landmark configurations was assessed using five randomly selected mandibles. To assess intraobserver variation, one researcher (AP) placed the landmarks and curve semilandmarks twice on each mandible with a week-long interval between landmarking sessions. To assess inter-observer variation, the set of landmarks was placed by an additional independent researcher (GA). To examine variations in shape, Principal components analysis (PCA) was carried out following a General Procrustes Analysis (GPA) of the landmark data, which eliminates differences in orientation, location, and size^37^. The significances of Procrustes distances within and between repeated measurements of specimens and by researchers were assessed via permutation tests (1000 random permutations)^38^.

#### Mandibular shape and muscle CSAs

For the 3D shape analysis, Cartesian coordinates were converted into shape variables through GPA. PCA was carried out to examine shape variation in the general population. Since mandibular size affects shape variation^19–21^ we controlled for allometry. Shape variables were regressed and standardized on centroid size (allometrically adjusted). A linear regression of log square roots of muscle CSAs on log centroid size (i.e., the independent variable) was used to allometrically adjust muscle CSAs.

Two-block Partial least squares (2B-PLS) analysis was carried out, separately for males and females, on allometrically adjusted muscle CSAs as one block and the adjusted shape variables as the second block, to examine the association between shape and muscle CSAs when allometry is accounted for. Visualization of shape changes along the PLS vector was carried out by warping the mean surface mesh using a triplet of thin plate splines (TPS) in the EVAN Toolbox (v. 1.71)^39^.

### Analysis of linear measurements

#### Reliability

Anthropometric measurement reliability was assessed using 15 randomly selected mandibles. To assess intraobserver variation in the linear and angular dimensions, a single researcher (TST) carried out the measurements twice with a two-week interval between each attempt. To assess interobserver error, measurements were taken by an additional independent researcher (HM or VS). Intraclass correlation coefficient (ICC) analysis was carried out to examine the reproducibility of the measurements and was interpreted according to the categorization method of Cicchetti^40^.

#### Mandibular linear measurements and muscle CSAs

A Kolmogorov-Smirnov test was carried out to test for the normality of distributions of the variables. Logarithmic transformation was carried out for variables that did not distribute normally. The association between muscle CSAs and mandibular measurements, both controlled for mandibular size (MGM), were assessed by calculating Pearson correlation coefficients. Data were controlled for sex (analyses were carried out separately for males and females) and age (using the partial correlation test).

### Data availability

The datasets analyzed during the current study are available from the corresponding author on request.

## Results

### Reliability analysis

Permutation tests of Procrustes distances indicated that differences in shape among repeated measurements of specimens were significantly greater than those among specimens, when landmarks were placed by the same researcher (*p* < 0.01). No significant differences in shape distances between researchers were found (*p* > 0.05). ICC results for the reproducibility of the linear, CSA and angular measurements showed good to excellent agreement (0.84≤ICC≤0.995 for intraobserver variation and 0.71≤ICC≤0.996 for interobserver variation)^40^.

### Mandibular shape variation

**Figure 4:**
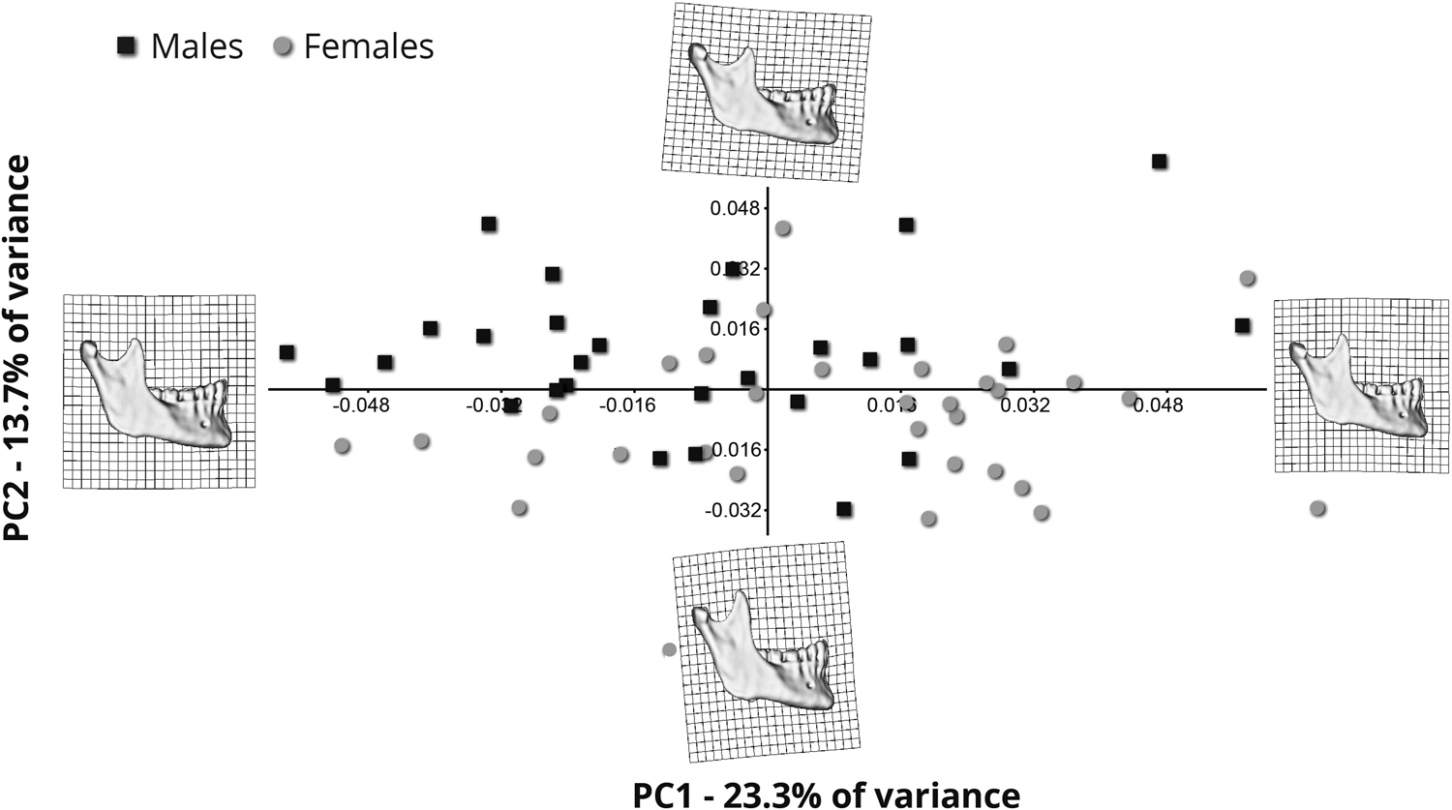
Principal component analysis of shape variation in the studied sample: Shape variables following general Procrustes analysis. The first two Principal Components (PCs) explain 37% of total variance.

37% of shape variation in the sample is explained by the first and second principal components of shape (PCs) (Fig. 4). Most female mandibles are located in the lower quadrants, whereas males are scattered mainly in the upper quadrants. The main aspect of shape variation represented by the first PC comprises changes in the shape of the mandibular body, which, warping along PC1, varies from being more triangular (right) to more rectangular (left). The main aspect of shape variation represented by the second PC relates to the ascending ramus which varies in shape between an elongated narrow parallelogram (lower) to a wide low trapezoid (upper), with the coronoid process varying in shape between an elevated-narrow-pointed structure to a low-wide-rounded one (Fig. 4).

### The association between mandibular shape and muscle CSA

**Figure 5:**
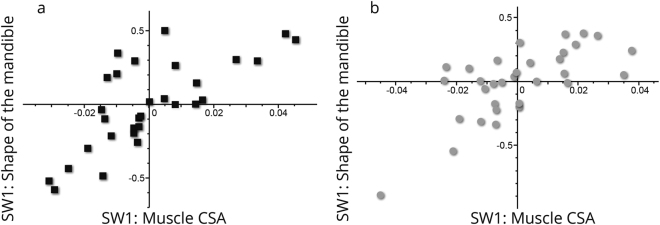
Plot of SW1 (mandibular shape) against SW1 (muscle CSA) from a two block partial least squares analysis in males (**a**) and females (**b**). Scores on these axes are significantly correlated (r = 0.734, p < 0.001 and r = 0.697, p < 0.001, respectively).

**Figure 6:**
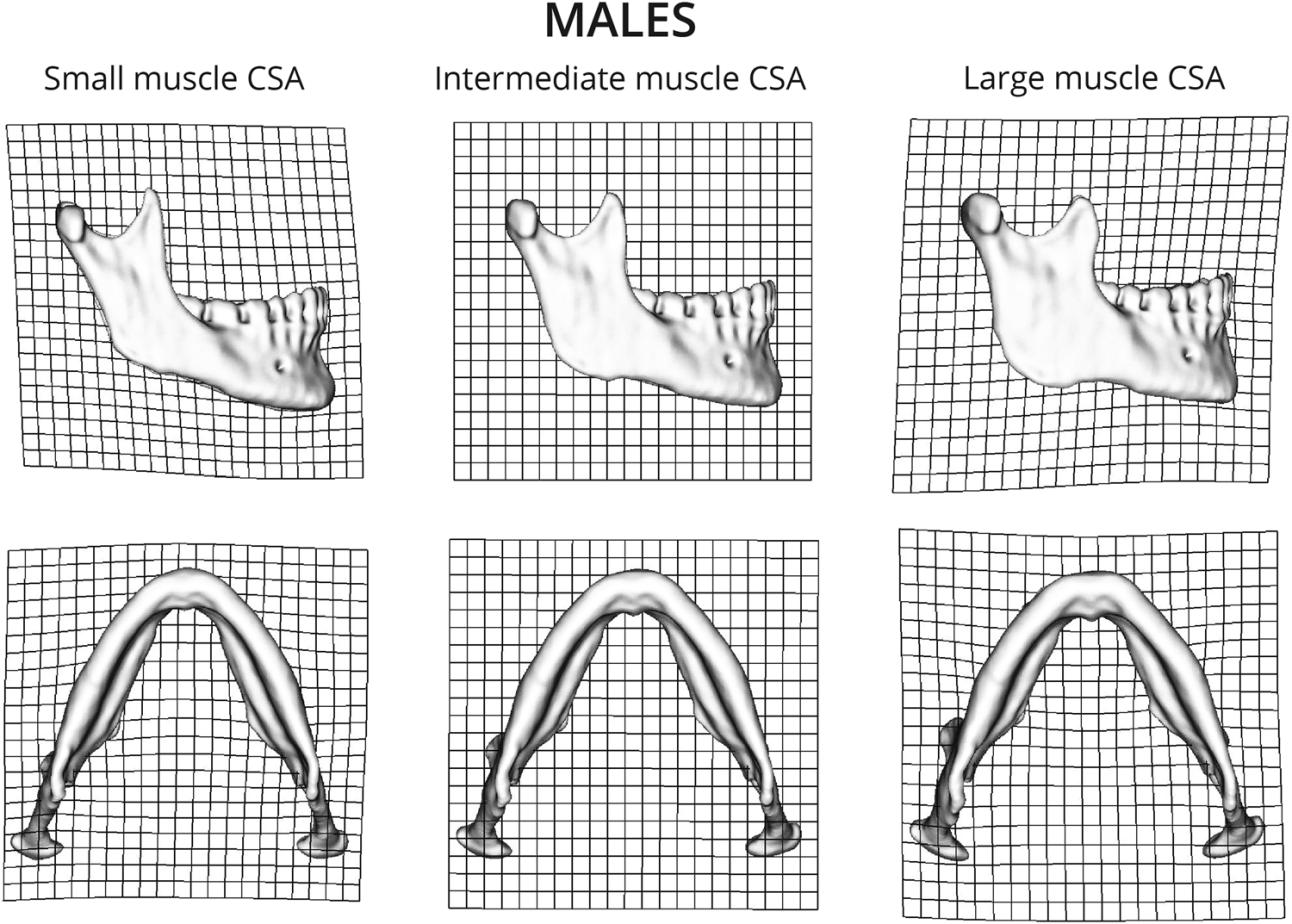
Warpings along SW1 of mandible shape in males. Large muscle CSAs are associated with a wider, more trapezoidal ramus, more massive coronoid, rectangular body and a more curved basal arch. Mandibles with smaller muscle CSAs are characterized by a tall and narrow ramus (more like a parallelogram) with a pointed coronoid, triangular body and a more triangular basal arch.

**Figure 7:**
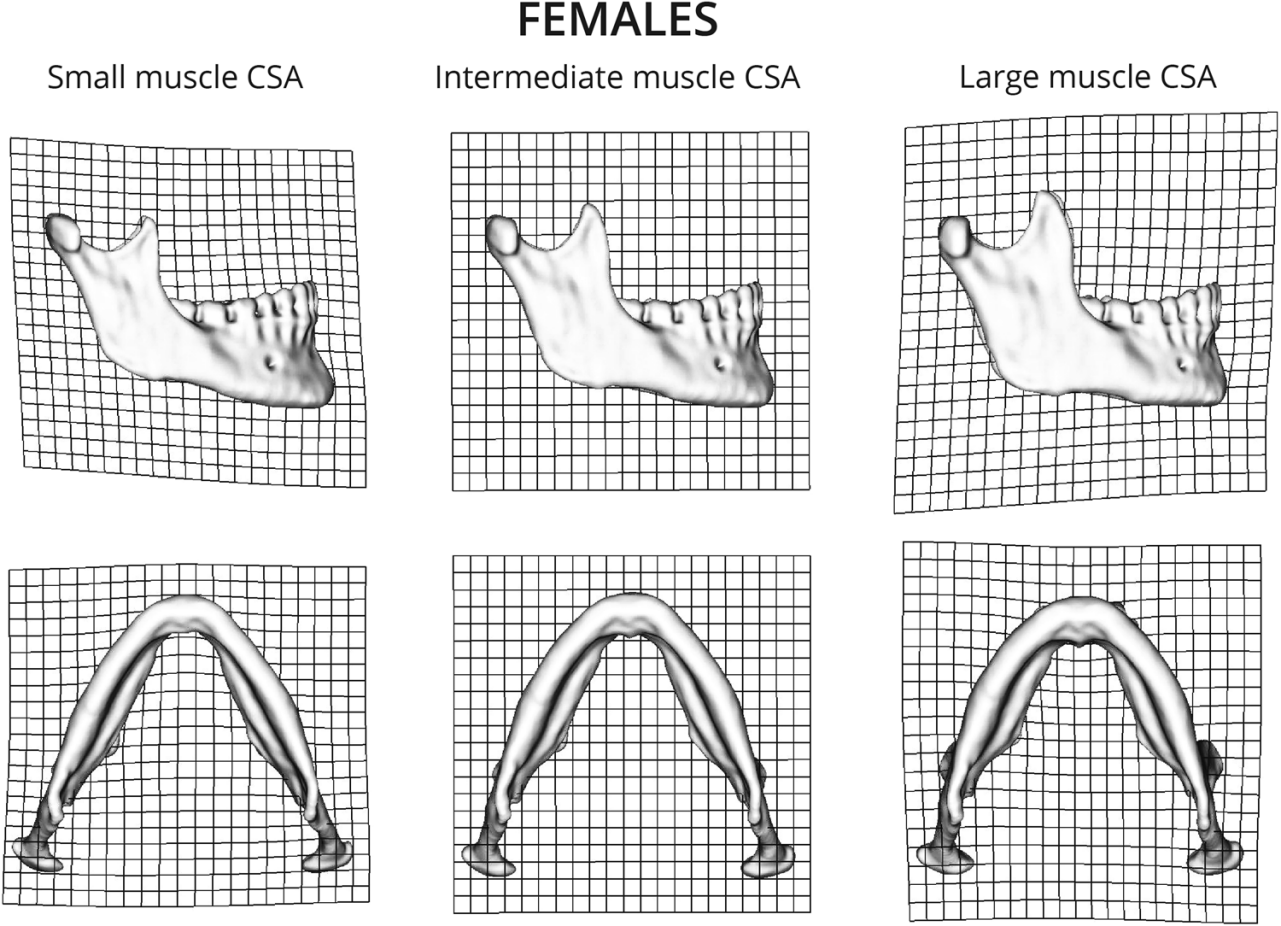
Warpings along SW1 of mandible shape in females. Large muscle CSAs are associated with a wider more trapezoidal ramus, more massive coronoid, rectangular body and a curved basal arch. Mandibles with smaller muscle CSAs are characterized by a tall and narrow ramus (more like a parallelogram) with a pointed coronoid, triangular body and a more triangular basal arch.

2B-PLS analyses between the first singular warps (SW1) of mandibular shape and the CSAs of the masseter and temporalis muscles, for both males and females, yielded high and significant correlations (r = 0.734, *p* < 0.001 and r = 0.697, *p* < 0.001, respectively) (Fig. 5). The visualization of the PLS for both males (Fig. 6) and females (Fig. 7) demonstrates that mandibles with large muscle CSAs manifest a wider more trapezoidal-shaped ramus, more massive coronoid, rectangular body and a curved basal arch. Mandibles with small CSA are characterized by a tall and narrow ramus (more like a parallelogram) with a pointed coronoid, triangular body and a more triangular basal arch.

### The association between mandibular metric characteristics and muscle areas

Associations between linear measurements and muscle CSAs controlled for MGM appear in Table 4. Most mandibular measurements manifested either significant, small correlations or no significant correlations with muscle CSAs (Table 4). This analysis has yielded three types of parameters: 1. Parameters not associated with muscle CSAs: mandibular angle, mandibular angle width, coronoid width, coronoid width CSA, body length, body height at premolars and its CSA. 2. Parameters associated with muscle CSAs in either males or females. For females: bigonial breadth (with masseter CSA). For males: mandibular angle CSA (with both muscle CSAs), ramus width and its CSA (with temporalis CSA), body height at molar (with temporalis CSA), body height CSA at molar (with both muscle CSAs) and symphysis thickness (with temporalis CSA) and chin height (with masseter CSA). 3. Parameters associated with muscle CSAs for both males and females: ramus length (with masseter CSA) and coronoid height (with both muscle CSAs).

**Table 4:** Partial correlations^1^ between masticatory muscle CSAs and mandibular measurements^$^.

Measurement	Masseter CSA		Temporalis CSA^#^	
	Males	Females	Males	Females
Bigonial breadth^#^	0.093	0.407^**^	0.050	0.099
Mandibular angle	−0.088	−0.028	0.053	−0.079
Mandibular angle width	0.126	0.003	0.168	0.068
Mandibular angle CSA	0.194^*^	0.033	0.307^**^	0.031
Ramus length	0.290^**^	0.280^**^	0.152	0.047
Ramus width	0.121	−0.078	0.214^*^	−0.035
Ramus width CSA	0.099	0.078	0.258^**^	0.039
Coronoid width	0.021	−0.131	0.082	0.044
Coronoid height	−0.350^**^	−0.272^**^	−0.282^**^	−0.130
Coronoid width CSA	0.055	0.097	0.173	0.148
Body length	0.048	0.093	−0.091	0.072
Body height at premolar	−0.065	0.018	0.003	−0.081
Body height at molar^#^	0.151	0.046	0.185^*^	−0.062
Body height at premolar CSA	0.127	0.134	0.127	0.010
Body height at molar CSA	0.211^*^	0.114	0.336^**^	0.032
Symphysis thickness	0.124	0.176	0.198^*^	−0.013
Chin height	0.189^*^	−0.048	0.062	−0.032

^1^Control for age.

^$^Muscle CSAs and mandibular measurements, except for mandibular angle, were controlled for mandibular size (MGM).

^#^Following logarithmic transformation.

^*^p < 0.05; **p < 0.01.

## Discussion

The current study shows that mandibular shape varies to a certain extent as a function of the forces applied to it by the temporalis and masseter muscles (Fig. 5). This is anticipated based on prior studies; “…the size and shape …of the jaws should reflect muscle size and activity”^13^ (p. 136). The major aspects of mandibular shape that covary with muscle CSAs, independent of sex, are, with larger CSAs, a wider trapezoidal ramus, a massive coronoid, a more rectangular body and curved basal arch. In contrast, mandibles with a tall and narrow ramus (parallelogram-like), a more pointed coronoid, a more triangular body and a more triangular basal arch were associated with smaller muscle CSA (Figs 6 and 7). In the absence of studies that directly measure the association between mandibular shape and masticatory muscle CSAs, our discussion is largely based on circumstantial evidence, namely, the associations between mandibular morphology and dental attrition (i.e., indicating extensive function of the masticatory muscles) and mandibular morphology and subsistence economy (i.e., softer diet requires less mastication force). For example, several anthropological studies have reported an association between excessive attrition and broad mandibles^41–44^. It has been shown that agriculturalists (softer diet) had relatively short and broad mandibles with a tall, angled ramus and coronoid process, whereas hunter-gatherer populations (harder diet) have relatively long and narrow mandibles with a short, upright ramus and coronoid process^24^. These results are in agreement with our observations.

Modern population studies offer similar insights. For example, individuals suffering from bruxism manifest broad mandibles^45–47^; subjects with strong bite forces tend to have a low mandibular plane angle and wide mandible, whereas those with weak bite force tend to have a high mandibular plane angle and narrow mandible^1,48,49^.

Direct evidence for an association between mandibular morphology and masticatory muscle force comes from clinical studies. For example, in individuals suffering from myotonic dystrophy of the masticatory muscles a greater mandibular angle and excessive vertical growth of the mandible was reported (e.g.^6,50^); and enlargement of the coronoid process was observed in individuals with temporalis muscle hyperactivity^51^.

Several animal experimental studies provide further support for this association. For example, pigs raised on a soft rather than a normal diet, manifested changes in jaw morphology and dental arch dimensions^52^; and reduced function of the masticatory system in rats caused changes in the width, height and thickness of the alveolar process and smaller cross-sectional area of the bone^16,17,53,54^.

Of the 17 linear parameters used in our study, 10 manifested significant low associations with muscle CSAs. Yet, these associations varied with sex and muscle (temporalis and/or masseter). Only two linear measurements (coronoid height and ramus length) showed significant, but weak, associations with muscle CSAs in both males and females when controlled for size. These results coincide with our shape analysis and highlight some of the biomechanical factors involved in mandibular design. For example, the anterior ramal border, from coronoid process downward, is under considerable tension during mastication^55^, potentially explaining the involvement of the temporalis and masseter muscles in shaping the ramus and coronoid. The increase in mandibular CSAs at the ramus, mandibular angle and body at the molar region, with muscle CSAs is in accordance with previous studies suggesting that the thickening and increase in height of the posterior part of the mandibular body with increased muscle strain is to enable the mandible to resist the parasagittal and transverse bending stresses, which are concentrated in these regions^56–59^. The idea of bone apposition over areas with increased demand to withstand bending force has been demonstrated in several human and animal studies (e.g.^16,17,60^).

Finally, all linear measurements in our study show, after correction for size, low correlations with muscle CSAs. This raises the question of why our findings do not support those of previous studies (e.g.^14,28,49^) that found high correlations. This might be because studies suggesting much higher correlations between mandibular linear measures and mastication force (e.g.^28,49^) did not correct their data for mandibular size. It is noteworthy that very few allometrically adjusted anthropometric variables show significant correlations with muscle CSAs. Those that do, largely reflect the findings of the PLS analyses of Figs 5–7 in that they measure ramus and coronoid form. However, given the strength of these associations they are likely useful only to predict the strength of masticatory muscle action among sample means rather than individuals. Indeed, the weak correlations shown by all variables stand in contrast to the PLS analyses of landmark data which find significant overall associations. This finding emphasizes the need to take a multivariate or landmark based approach to dietary retrodiction in archaeological populations. Even with such an approach, population loading history is most reliably inferred, rather than the diet or masticatory muscle force of any one individual.
